# Resistance of Undisturbed Soil Microbiomes to Ceftriaxone Indicates Extended Spectrum β-Lactamase Activity

**DOI:** 10.3389/fmicb.2015.01233

**Published:** 2015-11-10

**Authors:** Joao Gatica, Kun Yang, Eulyn Pagaling, Edouard Jurkevitch, Tao Yan, Eddie Cytryn

**Affiliations:** ^1^The Department of Soil and Water Sciences, The Robert H. Smith Faculty of Agriculture, Food and Environment, The Hebrew University of JerusalemRehovot, Israel; ^2^The Institute of Soil, Water and Environmental Sciences, The Volcani Center, Agricultural Research OrganizationBet-Dagan, Israel; ^3^Department of Civil and Environmental Engineering, University of Hawaii at ManoaHawaii, HI, USA; ^4^The Department of Plant Pathology and Microbiology, The Robert H. Smith Faculty of Agriculture, Food and Environment, The Hebrew University of JerusalemRehovot, Israel

**Keywords:** antibiotic resistance, undisturbed soils, soil bacteria, beta-lactamases, cephalosporins, ceftriaxone

## Abstract

Emergence and spread of antibiotic resistance, and specifically resistance to third generation cephalosporins associated with extended spectrum β-lactamase (ESBL) activity, is one of the greatest epidemiological challenges of our time. In this study we addressed the impact of the third generation cephalosporin ceftriaxone on microbial activity and bacterial community composition of two physically and chemically distinct undisturbed soils in highly regulated microcosm experiments. Surprisingly, periodical irrigation of the soils with clinical doses of ceftriaxone did not affect their microbial activity; and only moderately impacted the microbial diversity (α and β) of the two soils. Corresponding slurry experiments demonstrated that the antibiotic capacity of ceftriaxone rapidly diminished in the presence of soil, and ∼70% of this inactivation could be explained by biological activity. The biological nature of ceftriaxone degradation in soil was supported by microcosm experiments that amended model *Escherichia coli* strains to sterile and non-sterile soils in the presence and absence of ceftriaxone and by the ubiquitous presence of ESBL genes (*bla*TEM, *bla*CTX-M, and *bla*OXA) in soil DNA extracts. Collectively, these results suggest that the resistance of soil microbiomes to ceftriaxone stems from biological activity and even more, from broad-spectrum β-lactamase activity; raising questions regarding the scope and clinical implications of ESBLs in soil microbiomes.

## Introduction

The rapid emergence and spread of antibiotic resistance (AR) since the development of antibiotics in the 1940s, is one of the greatest epidemiological challenges of our time (Yang et al., 2010). The consequences of AR propagation in nosocomial and community settings include increased duration of hospital stays, higher healthcare costs and increased patient mortality (Foucault and Brouqui, 2007). According to Dhillon and Clark (2012), extended spectrum β-lactamases (ESBLs) are the major cause of hospital-acquired infections, particularly in intensive care units. They are also an important cause of failure of therapy with third generation cephalosporins, which are among the most widely used human and veterinary antibiotics in the world (Pitout, 2006; Perez et al., 2007; Falagas and Karageorgopoulos, 2009; Kummerer, 2009). ESBL-encoding genes in clinically-associated bacteria are generally situated on mobile elements such as plasmids, transposons and integrons, which can rapidly propagate on inter- and intra-species levels by means of horizontal gene transfer (Barnes et al., 1994; Robin et al., 2005; Watanabe et al., 2005).

Traditionally, the investigation of antibiotic resistant bacteria (ARB) has primarily focused on clinical environments (Brusetti et al., 2008); however, there is increasing evidence that AR is also common among environmental bacteria. A myriad of recent studies have detected antibiotic resistance genes (ARGs) with high sequence similarity to those found in clinical ARB in terrestrial environments distant from anthropogenic activities, and several studies have clearly shown that these genes far outdated the clinical use of antibiotics (Allen et al., 2009; D’Costa et al., 2011; Bhullar et al., 2012; Forsberg et al., 2014; Perron et al., 2015).

Soils are often subjected to anthropogenic influences such as wastewater and aquaculture discharge and manure and biosolid application that can introduce ARB and ARGs and result in propagation of natural AR due to selective pressure (Kummerer and Henninger, 2003; Binh et al., 2008; Davies and Davies, 2010; Knapp et al., 2010; Munir et al., 2011). However, the impact of anthropogenic practices on the soil resistome appears to be highly complex and ambiguous, as other studies have also reported cases where significant inputs of ARGs and ARB to soils by treated wastewater irrigation and manure did not significantly alter baseline levels of ARGs and ARB (McLain and Williams, 2010; Negreanu et al., 2012). Evidently, the high complexity of the soil matrix and the complex dynamics between soil, bacteria, and antibiotics significantly complicates understanding how anthropogenic activities influence AR in soil bacteria.

In this study we addressed the impact of the third generation cephalosporin ceftriaxone on microbial activity and bacterial community composition in two physically and chemically distinct undisturbed soils from Israel and Hawaii. Additionally, we evaluated ceftriaxone efficacy in soil microcosm and slurry experiments, and the presence of ESBL genes in soil microbiomes.

## Materials and Methods

### Soil Characteristics and Sampling

Four “undisturbed” (non-agricultural or human impacted) soils from Israel and Hawaii were used in microcosm and slurry experiments described in this study. Standard physical and chemical parameters and the geographical location of these soils are detailed in **Table 1**. Soil samples were taken from the field sites in triplicate by aseptically collecting ∼5 kg from the top 5 cm layer of soil, after brushing aside any loose litter using ethanol cleansed tools. The samples were placed in individual plastic bags, stored at 4°C and transported to the laboratory where they were immediately sieved and homogenized.

**Table 1:** Characteristics of soils used in this study.

Sampling site	Experiment	Clay (%)	Silt (%)	Sand (%)	Soil texture	pH	TN (%)	TOC (%)	Geographic coordinates
Gilat, Israel	Microcosms and slurries	16.25	13.75	70	Sandy loam	7.8	0.15	2.09	31°20′15.96″N 34°39′53.15″E
Carmel, Israel	Slurries	36.25	22.50	41.25	Clay loam	7.7	0.20	4.95	32°39′21.04″N 35°05′12.44″E
Leilehua, Hawaii	Microcosms	73.7	21.4	4.9	Clay	4.6	0.17	2.08	21°26′51.23″N 157°57′52.46″W
Manoa, Hawaii	Slurries	7.70	12.11	80.19	Loamy sand	5.5	0.12	1.9	21°20′18.82″N 157°48′00.24″W

*TN, total nitrogen; TOC, total organic carbon.*

### Bacterial Activity and Diversity Microcosm Experiments

#### Experimental Setup

Sandy loam and clay soils from Gilat (Israel) and Leilehua (Hawaii), respectively (**Table 1**) were distributed into triplicate plastic containers (300 g of soil each) and irrigated every 2 days to 50–60% water holding capacity with diluted nutrient broth (DNB, 0.08 g L^-1^) amended with ceftriaxone (2 μg/mL, Sigma). Concurrently, triplicate antibiotic-free microcosms were irrigated with just DNB. The microbial activity and community structure of the two soils, with and without ceftriaxone amendment, was measured at 0, 7, and 14 days subsequent to experimental initiation, as described below. To ensure drainage, 5 mm diameter holes were punched at the bottom of the microcosm containers, and the bottom of the containers were lined with a layer of sterile tuff (volcanic gravel- diameter of 10–20 mm) covered by a sterile polyethylene net with a mesh size of 1.2 mm; upon which the soil was added.

#### Determination of Soil Microbial Activity

Soil samples taken at the above mentioned time points were used to determinate soil microbial hydrolytic activity estimated with the fluorescein di-acetate (FDA) method previously described by Casida et al. (1964). Briefly, 5 g of soil from each microcosm was mixed in a 100 ml Erlenmeyer flask with 45 ml of 0.2 mol of phosphate buffer, pH 7.6; subsequently, 100 μl of 3,6-diacetylfluorescein solution was added, and each flask was incubated for 30 min in a rotary shaker at 30°C. Soil was then removed from the mixture by centrifugation at 6000 rpm for 5 min. Finally, the absorbance of the reaction product, fluorescein was spectroscopically measured at 494 nm.

In parallel, soil oxidative activity was estimated using the dehydrogenase assay according to Schurer and Rosswall (1982). In 50 ml Erlenmeyer flasks, 6 g of each soil was mixed with 0.2 g of CaCO_3_, 2.5 ml of distilled water and 1 ml of 3% aqueous solution of 2,3,5-triphenyl tetrazolium chloride (TTC). Samples were incubated at 37°C for 24 h. The soil reaction was stopped by the addition of 10 ml of ethanol and shaking for 1 min. Finally, soil solutions were filtered and absorbance of the product, triphenylformazan (TPF) was spectroscopically measured at 485 nm.

Determination of statistical differences between treatments for both assays was performed by non-parametric tests (Kruskal–Wallis) in SPSS 18.0.0 (SPSS, Inc., Chicago, IL, USA); *p*-values of less than 0.05 were considered to be significant.

#### DNA Extraction and High Throughput Sequencing

DNA was extracted from the microcosm samples with the PowerSoil DNA Isolation kit (MoBio, Laboratories, Carlsbad, CA, USA) using the protocol described by the manufacturer, and amplified with the universal bacterial 16S rRNA gene primers 530F and 1100R as previously described (Dowd et al., 2008a,b). Subsequently, amplicons were subjected to bacterial tag-encoded FLX amplicon pyrosequencing (bTEFAP) at the Research and Testing Laboratory (RTL-Lubbock, TX, USA) using protocols available on the RTL website (www.researchandtesting.com).

### Bioinformatics and Statistical Analyses of 16S rRNA Gene Amplicons

Bioinformatic analysis of pyrosequencing data applied the Quantitative Insights into Microbial Ecology (QIIME; version 1.7.0) pipeline (Caporaso et al., 2010b). Sequences with: a quality score under 25, nucleotide length that was not between 200 and 1000 bp, more than six ambiguous bases, and mismatches in primer and barcode sequences, were not used for downstream analysis.

After barcode and primer sequence removal, redundant sequences were clustered into operational taxonomic units (OTUs; 97% similarity cutoff) using UCLUST (Edgar, 2010). Representative sequences were chosen for further analysis based on the most abundant sequence criteria. These sequences were aligned using PyNAST with a minimum percent identity of 97% (Caporaso et al., 2010a). Taxonomy assignment was performed using the Ribosomal Database Project (RDP) Naive Bayes classifier. Finally, chimeras were identified and removed using ChimeraSlayer (Haas et al., 2011) and OTU singletons were also removed from the analysis.

Operational taxonomic units generated from the data processing were used to determinate α and β diversity and diversity indexes for each soil. For β diversity, OTUs were pooled using 80% similarity thresholds, following subsampling of data using the total number of sequences in the smaller sample (2076 and 1812 sequences/sample for the Israeli and Hawaiian soils, respectively). Pairwise comparisons between samples in each dataset (Gilat and Hawaii) were performed using unweighted and weighted UniFrac analysis (Lozupone and Knight, 2005). Statistical differences between groups of samples were tested by PERMDISP (Anderson et al., 2006) available through QIIME. Additionally, ordinations were plotted using non-metric multidimensional scaling (NMDS) of Bray–Curtis distance matrices from each soil, using the 25% most abundant OTUs. Finally, an indicator species analysis with Monte Carlo test of significance was performed to identifying OTUs that showed strong preferential distributions with respect to specific treatment using the Multivariate Analysis of Ecological Data (PC-ORD 5.32; MjM, USA) according to the procedure described by Pasternak et al. (2013).

Determination of statistical differences of both diversity indexes and taxonomic groups were performed by non-parametric tests (Kruskal–Wallis) in SPSS 18.0.0 (SPSS, Inc., Chicago, IL, USA), where a *p*-value of less than 0.05 was considered to be significant.

### Assessment of Ceftriaxone Efficacy in Soil

#### Slurry Experiments

Triplicate sterile and non-sterile slurries were prepared from Gilat, Carmel, and Manoa soils (**Table 1**). Sterilized soils were autoclaved three times at 120°C with 24 h intervals between each sterilization. In all of the treatments, 5 g of sieved and homogenized soil was added to Erlenmeyer flasks together with 45 mL of DNB (0.08 g L^-1^) and ceftriaxone (100 μg/mL final concentration). The slurries obtained were incubated at 37°C with agitation at 180 rpm for 2 weeks. The ceftriaxone efficacy in soil slurries was assessed at periodical intervals by measuring the inhibition zone diameter generated following application of slurry filtrates onto *Escherichia coli* spread plates. Briefly, 100 μL of ceftriaxone-susceptible *E. coli* DH5α, cells (OD_600_ = 0.6–0.7) were spread onto LB agar plates in triplicates. At each time interval, 500 μL of the liquid fraction from each Erlenmeyer flask was filtered through a 0.2 μm membrane and 5 μL of filtrate was applied to the center of fresh *E. coli* spread plates, which were then incubated at 37°C for 24 h. In tandem, 5 μL of water was applied to plates as a negative control. The relative antibiotic efficacy (%) for each treatment was presented as the inhibition zone diameter at a particular time relative to the inhibition zone measured at time zero.

#### Microcosm Experiments

To further evaluate the *in-terra* ceftriaxone efficacy, we applied additional microcosm experiments using sterile (three 50-min autoclave cycles separated by 24 h intervals) and non-sterile Gilat soil (**Table 1**) with and without ceftriaxone amendment (four different treatments altogether). *E. coli* sp. cv601 cells, susceptible to ceftriaxone but resistant to 50 μg mL^-1^ of rifampicin and 25 μg mL^-1^ of ampicillin (Smalla et al., 2000), were grown in Luria-Bertani (LB) broth, harvested by centrifugation, washed three times with phosphate buffer solution (10 mM, pH 7.2) and resuspended and adjusted with sterile deionized water to achieve a concentration of ∼1 × 10^8^ colony-forming units per milliliter (CFU mL^-1^). Sterile and non-sterile soils were then inoculated with approximately 2500 *E. coli* cells per gram of soil, deposited into plastic containers in triplicates and incubated in the dark at 30°C. Microcosms were irrigated after 3 h with DNB (0.08 g L^-1^) with or without ceftriaxone (2 μg ml^-1^), and subsequently, every 2 days to 50–60% of the soil water holding capacity in the following treatments: (i) sterile soil amended with ceftriaxone (SA+), (ii) sterile soil not amended with ceftriaxone (SA-), (iii) non-sterile soil amended with ceftriaxone (NA+), and (iv) non-sterile soils not amended with ceftriaxone (NA-). For quantification of *E. coli* cells, soils were sampled at different time points, serially diluted in saline medium (9 g L^-1^ of CaCl), plated on LB agar amended with rifampicin (10 μg mL^-1^) and incubated for 24 h at 37°C.

#### PCR Detection of ESBLs Genes

Polymerase chain reaction (PCR) amplification of selected ESBL (*bla*TEM, *bla*CTX-M, *bla*OXA, *bla*SHV) and metalo-β lactamase (*bla*VIM and *bla*NDM) genes (with β-lactamase activity toward ceftriaxone) was performed on DNA extracted from all three time points of the initial non-amended and antibiotic-amended microcosms. The touchdown PCR protocols for the *bla*OXA and *bla*SHV genes were performed with modifications according to Bert et al. (2002) and Paterson et al. (2003). Touchdown reactions for *bla*TEM and *bla*VIM genes were based on the protocols of Monstein et al. (2007) and Mazzariol et al. (2011). Amplification of *bla*CTX-M gene was achieved using a modified version of the Pitout et al. (2004) protocol; and finally, amplification of the *bla*NDM gene was based on the Nordmann et al. (2011). PCR protocols and primer details are presented in Supplementary Table S6. All PCR reactions were performed in a final volume of 50 μL containing 1.25 U Taq DNA polymerase and 5 μL of Taq buffer (DreamTaq; MBI Fermentas, Lithuania), 1.5 mM of MgCl_2_, 200 μM of dNTPs, 0.2 μM of each primer and DNA template at concentration of 20 ng. PCR amplicons were visualized in 1% agarose gels stained with ethidium bromide. Representative positive results of PCR reactions were cloned into pGEM-T easy vectors (Promega), transformed into competent *E. coli* DH5α cells and sequenced using an ABI Prism 3100 genetic analyzer (Applied Biosystems, Foster City, CA, USA) for ESBL gene confirmation.

#### Accession Numbers

The sequence data from this study have been deposited in the DNA Data Bank of Japan under accession numbers DRA001180 and DRA001181.

## Results

### Soil Microbial Activity

**FIGURE 1:**
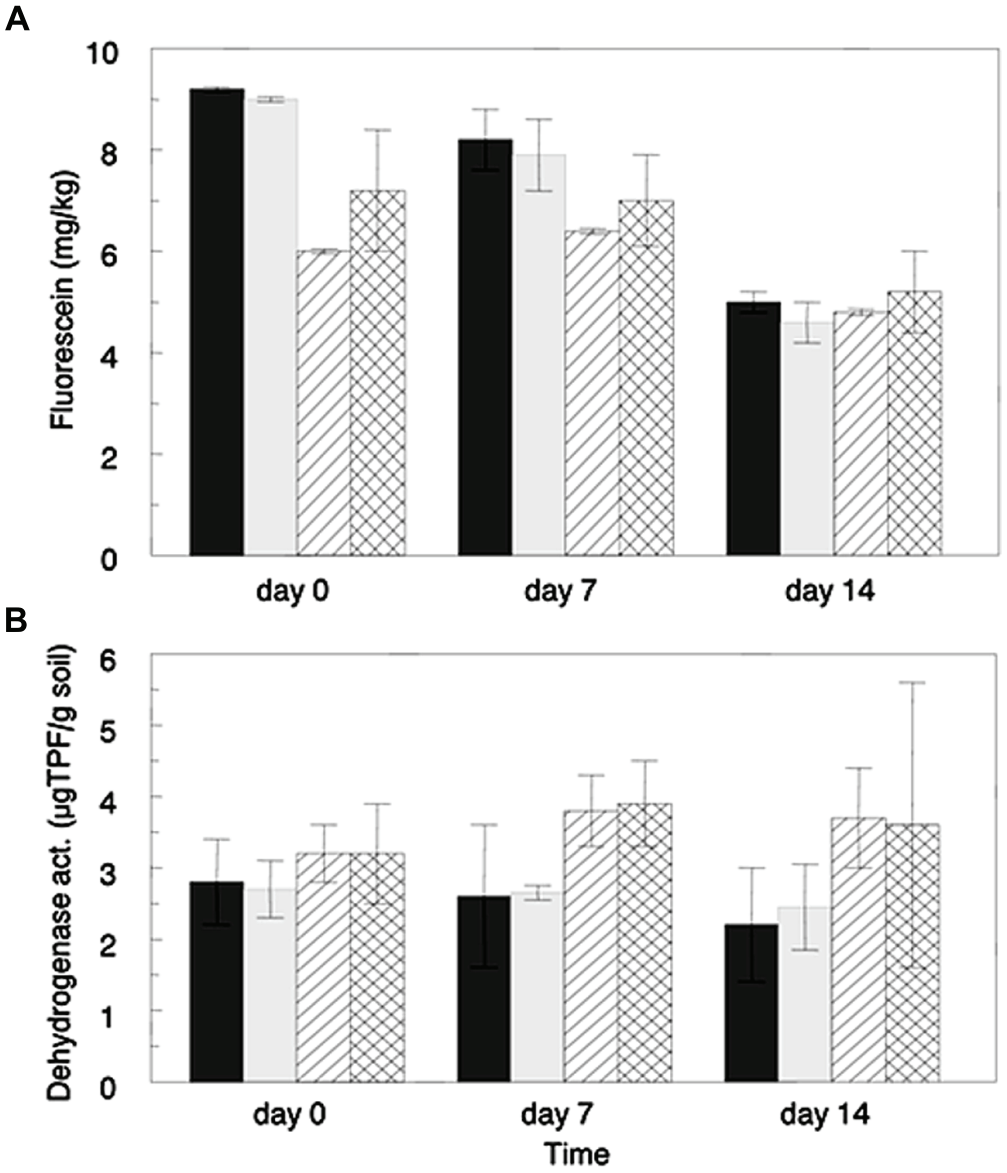
**Microbial activities in soils amended with ceftriaxone (2 μg/mL) and in corresponding non-amended soils estimated by the Fluorescein di-acetate hydrolysis assay (A), and by the dehydrogenase assay (B).** Values represent averages of three microcosm replicates. Sandy loam soils (Gilat) are represented by line filed columns (diagonal lines for non-amended and perpendicular lines for antibiotic treatments) and clay soils are represented by gray columns (dark gray for non-amended and light gray for antibiotic-amended treatments). Bars represent standard deviation average of each treatment.

Microbial hydrolytic activity and oxidative metabolic activity in microcosm experiments were estimated using the FDA hydrolysis and dehydrogenase assays, respectively. Hydrolytic activity was initially higher in the Hawaiian acid clay soil than in the sandy loam soil from Israel (**Figure 1A**); however, after 2 weeks the activity of the two soils was very similar. A gradual diminution in the hydrolytic activity was observed in the clay soil from almost 9 mg fluorescein kg^-1^ at the beginning of the experiment to ∼5 mg of fluorescein kg^-1^ on day 14 (significant differences between time zero and time 14 days, *p* = 0.027). In contrast, the sandy loam soil showed similar levels of hydrolytic activity between the beginning of the experiment and day 7, and a slight reduction (no statistical significance) in the last week of the experiment. Interestingly, no significant differences were found in the hydrolytic activity of the non-amended relative to the antibiotic-amended microcosms for either of the soil types at any of the sampled time points analyzed.

Oxidative metabolic activity levels (**Figure 1B**), were generally higher in the sandy loam soil than the clay soil; however, these differences were not significant. Additionally, the metabolic activity of both of the clay soil microcosm treatments slightly decreased over the 2 weeks experiment. In contrast, in the sandy loam microcosms the metabolic activity increased during the first week of incubation, and slightly decreased in the week thereafter. As well as the hydrolytic activity, no significant differences were observed in levels of metabolic activity of the ceftriaxone-amended microcosms in relation to the non-amended ones, for either of the analyzed soils at any time point, supporting the FDA results described above.

### 16S rRNA Gene Amplicon Sequence Analysis

**FIGURE 2:**
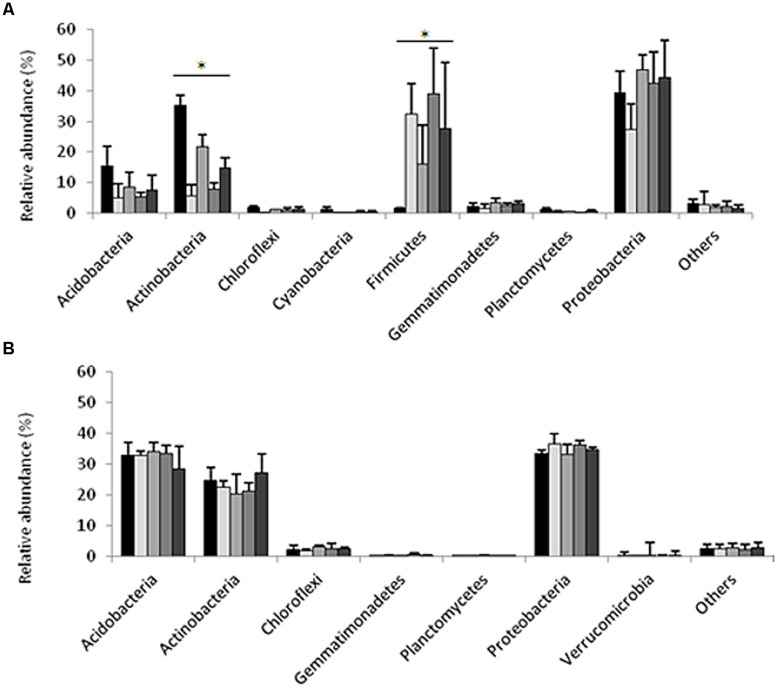
**Relative abundance of dominant phyla generated by pyrosequencing of 16S rRNA gene amplicons from the sandy loam (A), and clay (B) soil microcosms.** The first bar in each phylum indicates samples taken at time zero; the second and third bars indicate samples taken at 7 days, non-amended and antibiotic-amended treatments respectively; and the fourth and fifth bars indicate samples taken at 14 days, control- and antibiotic-amended treatments respectively. Asterisks indicate significances differences between treatments for a specific phylum. Bars represent average standard deviations of each treatment.

Pyrosequencing of 16S rRNA gene amplicons from the sandy loam and clay soil microcosms (*n* = 15 per site) generated a total of 51,882 and 65,033 sequences, respectively. The average length of the sequences was 400 bp for the sandy loam samples and 351 bp for the clay soil samples. Quality control screening and binning resulted in a total of 2,479 and 1,886 unique OTUs for the sandy loam and clay soils, respectively. The number of sequences obtained from the individual sandy loam and clay soil microcosms ranged from 2,089 to 4,721 and 2,012 to 7,033, respectively, with corresponding mean values of 3,395 and 4,160 sequences per soil sample. The relative abundances of major bacterial phyla in the original soils, in the ceftriaxone-amended and ceftriaxone non-amended sandy loam and clay microcosms, one and 2 weeks following experimental initiation, are shown in **Figure 2**. *Proteobacteria* was the major phylum in all of the sandy loam and clay soil microcosms, with relative abundances of 27.2–46.8% and 33.1–36.7% of the total defined phyla, respectively. *Actinobacteria* were also highly abundant in both soils with relative abundances of 5.7–35.2% and 20.5–27.2% for the sandy loam and clay soil microcosms, respectively. *Acidobacteria* also represented a significant fraction of the defined phyla, but values in the clay soils were significantly higher than in the sandy loam soils (28.7–34.2% vs. 4.9–15.3%). The relative abundance of *Firmicutes* was low in the initial sandy loam soil sample (1.3%) but increased (16–39%) in non-amended and antibiotic amended soils after 7 and 14 days. In contrast, the relative abundance of *Firmicutes* in the clay soil was below the detection limit (0.1%) in any of the treatments analyzed. In general, the bacterial community composition appeared to be more dynamic over time in the sandy loam soil microcosms than in the clay soil microcosms.

Non-parametric statistical analyses were applied in order to identify specific taxa whose relative abundances were significantly impacted (induced or repressed) by ceftriaxone amendment. In the sandy loam microcosms, the relative abundances of the *Actinobacteria* were higher in antibiotic amendment soils than in non-amended soils (Kruskal–Wallis test, *p* = 0.012; Supplementary Table S1); in contrast, the relative abundance of *Firmicutes* was lower in the antibiotic amended soils than non-amended soils (Kruskal–Wallis test, *p* = 0.042). However, these differences are principally produced by the relative abundance of *Actinobacteria* and *Firmicutes* at time zero in comparison with other time points. No significant differences (Kruskal–Wallis test, *p* > 0.05) were observed in the clay soils, which showed very slight changes across the different phyla and treatments. The same analysis was performed at different taxonomic levels such as class, order and family (data not shown) in the two soils; however, pairwise comparisons failed to show any significant differences between non-amended and ceftriaxone-amended treatments for a given time point (7 or 14 days).

**FIGURE 3:**
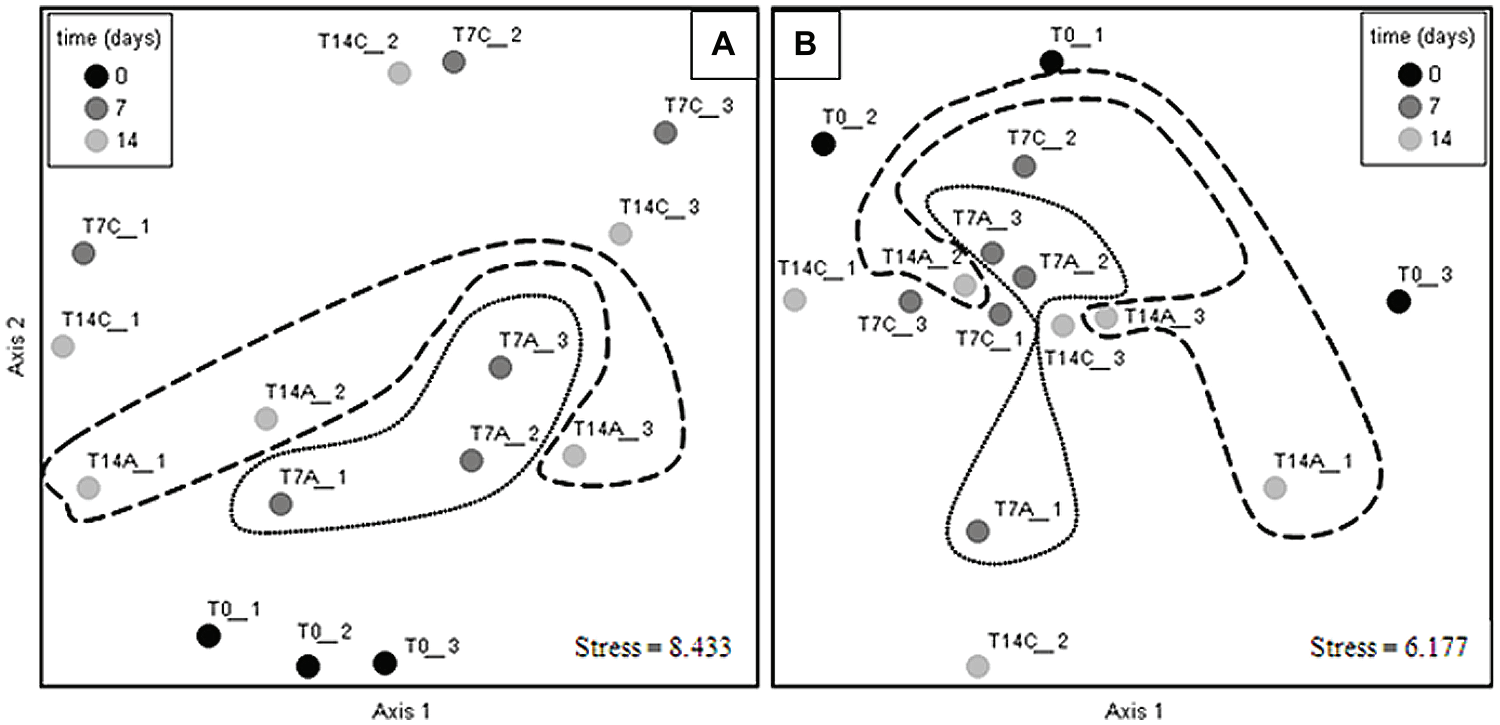
**Non-metric multidimensional scaling (NMDS) ordination of bacterial communities in the sandy loam (A), and clay (B) soil microcosms.** Closer points indicate more similar communities. Antibiotic treatments are circled with dotted lines for 7 days microcosms and with dashed lines for 14 days microcosms; Ordination axes have no biological meaning. Circles in the NMDS plots were added to facilitate the visualization of the different treatments and does not represent significant differences between controls and antibiotic treatments. Non-amended control microcosms (C), antibiotic-amended microcosms (A); experimental initiation (T0- black circles), 7 days after experimental initiation (T7- dark gray circles), 14 days after experimental initiation (T14- light gray circles). Numbers at the end of sample’s name indicate the replicate number.

Alpha diversity indexes were tested across the different treatments (**Table 2**) in each soil analyzed; the statistical analysis results show no significant differences (Kruskal–Wallis, i > 0.05) when comparing non-amended vs. antibiotic amended treatments from either of the soils at 7 or 14 days subsequent to experimental initiation (Supplementary Table S2). These results were supported by β diversity analyses, which showed that antibiotic irrigation did not have significant effects on the bacterial composition at the community scale. The lack of differences between antibiotic treatments is observable by NMDS of an averaged Bray–Curtis distance matrix; thus, the NMDS of each soil analyzed showed that the samples did not cluster according to treatment or time; and hence, the ceftriaxone amendment did not affect the bacterial community profile in either of the soils analyzed (**Figure 3**). The same phenomenon was observed when cluster analysis was performed (Supplementary Figure S1).

**Table 2:** Diversity indexes of original soil and microcosm microbiomes based on pyrosequencing analysis of 16S rRNA gene amplicions at 3% dissimilarity.

	Sandy loam soils					Clay soils				
Treatment	T0	T7C	T7A	T14C	T14A	T0	T7C	T7A	T14C	T14A
Taxa	702.33	585.33	680	451	458	527	711.33	688.66	491	527.66
Simpson	0.986	0.978	0.980	0.981	0.971	0.983	0.989	0.985	0.985	0.984
Shannon	5.58	4.98	5.31	5.01	4.99	5.15	5.48	5.33	5.191	5.20
Evenness	0.384	0.251	0.303	0.339	0.330	0.342	0.342	0.306	0.371	0.354
Chao1	784.70	693.03	799.46	515.80	503.70	621.3	849.13	827.13	581.46	593.36

*T0, experimental initiation; T7, 7 days after experimental initiation; T14, 14 days after experimental initiation; C, Non-amended control microcosms; A, antibiotic-amended microcosms.*

Statistical comparisons of the bacterial profiles in the different microcosms were performed using PERMDISP on QIIME (Supplementary Table S3). None of these analyses showed any significant time- or ceftriaxone-related differences in community composition in either of the analyzed soils. Moreover, the core microbiome of soils for each treatment remained quite similar at the phylum (Supplementary Figures S2 and S3), class and order levels (data not shown) with two exceptions in the sandy loam soils: the *Firmicutes*, whose relative abundance decreased in ceftriaxone-irrigated microcosms at both 7 and 14 days; and *Actinobacteria* whose relative abundance increased in soils amended with ceftriaxone for both times tested (7 and 14 days). In order to identify specific OTUs whose relative abundances were significantly impacted (induced or repressed) by ceftriaxone amendment, an indicator species analysis was performed in PC-ORD. Very few OTUs showed preferential distribution in response to antibiotic amendment (12.2% of OTUs in the sandy loam soils, and 5.3% of the OTUs in the clay soils); and in these cases, frequently OTUs that were significantly more abundant in the antibiotic-amended microcosms belonged to the same families as OTUs that were more abundant in the non-amended samples (Supplementary Tables S4 and S5), indicating that ceftriaxone resistance was not specifically linked to distinct bacteria phyla.

### Ceftriaxone Efficacy in Soil Slurries

**FIGURE 4:**
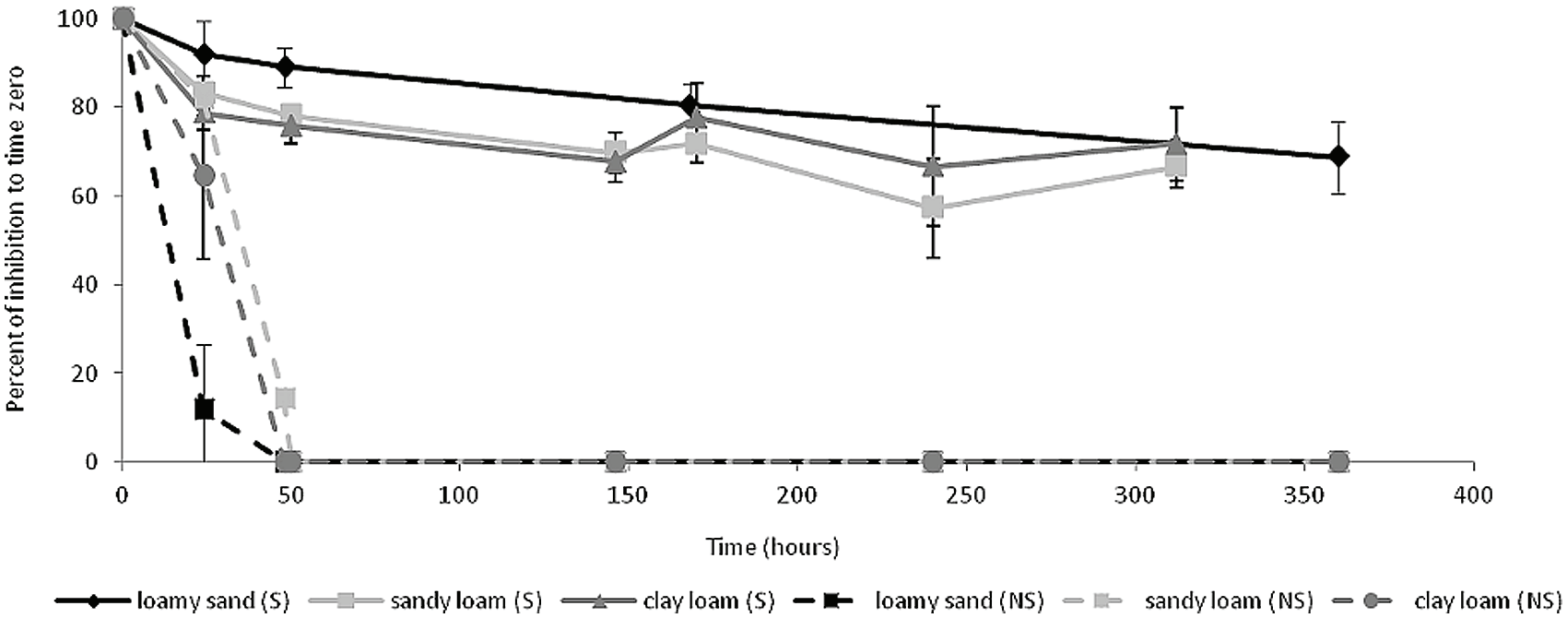
**Ceftriaxone activity in soil slurries.** Black lines (Manoa soil), dark gray lines (Carmel soil) and light gray lines (Gilat soil). Soils are grouped into sterile soils (S, continuous lines) and non-sterile soils (NS, dashed lines).

The efficacy of ceftriaxone was evaluated in three physicochemically-diverse soils (Gilat, Carmel, and Manoa, **Table 1**) by incubating ceftriaxone in sterilized and non-sterilized soil slurries for a period of 14 days and testing the potency of the slurry filtrates on *E. coli* coated plates as described in the materials and methods section. In the non-sterile slurries, the efficacy of ceftriaxone rapidly decreased, and slurry filtrates were almost completely inactivated after 48 h subsequent to experimental initiation (**Figure 4**). In contrast in all the sterilized soils, ceftriaxone efficacy was relatively stable and remained ∼70% of that of the initial inoculation until the end of the experiment (14 days).

### Ceftriaxone Efficacy in Soil Microcosms

**FIGURE 5:**
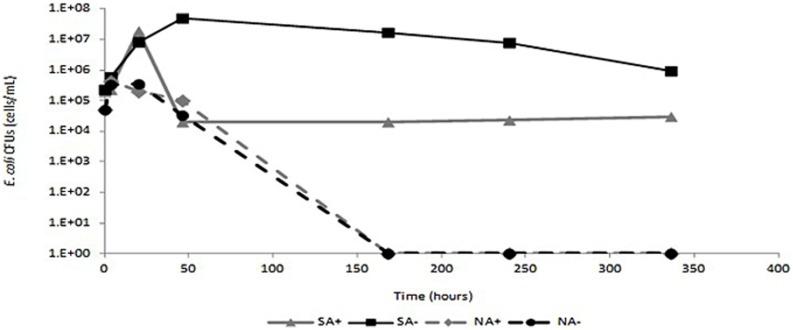
**Survival of *Escherichia coli* in sandy loam soil.** Black line- non-amended soils; gray line- ceftriaxone-amended soils. Sterile soils are indicated by continuous lines and non-sterile soils are indicated by dashed lines. SA+ indicates sterile ceftriaxone-amended soil; SA- indicates sterile non-amended soils; NA+ indicates non-sterile ceftriaxone-amended soils; NA- indicates non-sterile non-amended soils.

To further assess the *in-terra* antibacterial efficacy of ceftriaxone, sterile and non-sterile sandy loam microcosms were amended with *E. coli* cells, irrigated with and without ceftriaxone and incubated for 336 h as described in detail in the materials and methods section. In sterile microcosms not amended with ceftriaxone (SA-), *E. coli* abundance reached levels of almost 4.9 × 10^7^ CFU g^-1^ soil and remained relatively stable for the duration of the experiment, slightly decreasing only after 336 h of incubation (**Figure 5**). Conversely, in sterile microcosms amended with ceftriaxone (SA+), *E. coli* abundance initially reached 1.9 × 10^7^ CFU g^-1^ soil, but rapidly diminished (within 50 h) to baseline levels (2.3 × 10^4^ CFU g^-1^ soil), and remained at these levels for the duration of the experiment. In contrast, the *E. coli* survival profiles were identical in the antibiotic-amended (NA+) and non-amended (NA-) microcosms, where both were characterized by an initial proliferation in *E. coli* abundance within the first 20 h reaching levels of ∼4.3 × 10^5^ and 3.3 × 10^5^ CFU g^-1^ soil respectively, followed by a dramatic decline after 46 h of incubation, where *E. coli* values were below detection limits. Collectively, these results indicate that ceftriaxone is biologically active in the soil microcosms.

### Presence of ESBL Genes in Soils

The results obtained in microcosm and slurry experiments show that the ceftriaxone efficacy rapidly diminished in non-sterilized soils. As previously noted, ESBLs are an important and broad group of bacterial enzymes responsible for resistance to β-lactams. We screened the Gilat (sandy loam) and Manoa (clay) soils (**Table 1**) for presence of four clinically-relevant ESBL gene families as a potential explanation for the observed *in-terra* reduction in ceftriaxone efficacy. The results showed that *bla*TEM genes were ubiquitous to all of the clay and sandy loam microcosms analyzed. The amplified *bla*TEM fragment is highly conserved and therefore it was not possible to determine the precise classification of these genes based on sequencing of the cloned amplicons. Unlike *bla*TEM, *bla*CTX-M was detected in the sandy loam soil microcosms but not in the clay soil from Hawaii. Cloning and sequencing showed that the detected sequences were most closely related to *bla*CTX-M 15 (99% similarity). In addition, an amplicon with partial identity to *bla*OXA-1 from *Salmonella enterica* subsp. enterica serovar was identified in the Hawaiian soil. *bla*SHV. The metalo- β-lactamase genes *bla*VIM and *bla*NDM were not detected in the sandy loam or clay soils analyzed.

## Discussion

β-lactamases that degrade third generation cephalosporins, are perhaps the most important AR mechanisms in bacterial pathogens; however, little is known about the scope and activity of these enzymes in soil microbiomes. In this study, we applied a series of soil microcosm and slurry experiments to determine the impact of third generation cephalosporins on microbial dynamics, and to elucidate the scope of β-lactamase activity in soil.

Initially, the impact of third-generation cephalosporin amendment on soil bacterial community composition and microbial activity was assessed in a series of controlled microcosm experiments in undisturbed soils with two different soil types (clay and sandy loam) from geographically distant areas of the world (Israel and Hawaii). Surprisingly, despite the fact that soil microcosms were irrigated every 2 days with clinically-relevant concentrations of ceftriaxone, known to have broad-spectrum activity against Gram-positive and Gram-negative bacteria, no significant differences in levels of microbial activity were observed when non-amended treatments were compared to antibiotic-amended treatments.

The insignificant impact of ceftriaxone on soil microbial activity is congruent with the analysis of 16S rRNA gene amplicons, which revealed that very few taxonomic groups were consistently susceptible to irrigation with ceftriaxone (Supplementary Tables S4 and S5), and no significant change in α and β diversity between control and antibiotic-amended soils at individual time points were observe (**Figures 1–3**). Thus, *Acidobacteria*, *Actinobacteria*, and *Proteobacteria* were the most abundant phyla in both of the analyzed soils; characteristic of the community composition in comparable soils analyzed in previous studies (Kim et al., 2012; Rampelotto et al., 2013). Although no significant changes were detected between treatments, a clear temporal trend in community composition was observed in the sandy loam soil. Seven days after the experimental initiation revealed changes in the relative abundance of *Firmicutes* and *Actinobacteria* which increased and decreased, respectively in both the amended and non-amended samples. These results are similar to previously reported changes in the relative abundance of the same phyla in semiarid grassland after the beginning of the rainy season (McHugh et al., 2014). Interestingly, these changes in relative abundance are not observed in the Hawaiian soil, where natural soil moisture levels are generally higher than Israeli soils. Hence, the changes observed in our results can be related to the irrigation process applied in our experiment. In addition, the higher relative abundance of *Acidobacteria* in the clay soil can be explained by the low pH in these volcanic soils (pH 4.6 vs. pH 7.8 in the sandy loam soil), which is supported by previous studies that have demonstrated that reduced pH favors the growth of *Acidobacteria* (Jones et al., 2009; Dimitriu and Grayston, 2010; Rampelotto et al., 2013).

Collectively, the results of the biochemical and phylogenetic analyses described above, suggest that the clinically-relevant concentrations of ceftriaxone amended to the soil microcosms were inactivated by either chemical interactions in the soil (such as sorption), or soil microbial-activity (probably associated to β-lactamase activity, the primary mechanism associated with ceftriaxone resistance). To test these hypotheses, we measured the antibacterial activity of ceftriaxone in soil slurries (**Figure 4**) and monitored the dynamics of an introduced *E. coli* strain in sterile and non-sterile microcosms in the presence and absence of ceftriaxone (**Figure 5**). In the slurry experiments, the 20–30% reduction in ceftriaxone efficacy observed in the sterile slurries during the 2 weeks incubation period (**Figure 4**), seemingly stemmed from sorption or additional *in-terra* physiochemical mechanisms. In contrast, the rapid and complete inactivation (within 48 h) of ceftriaxone in the non-sterile slurries strongly suggests that most (70–80%) of the ceftriaxone inactivation was associated with biological activity. This is supported by the three orders of magnitude reduction in *E. coli* abundance in the ceftriaxone-amended vs. non-amended sterile soil microcosm experiments and by the PCR-based detection of *bla*TEM, *bla*OXA, and *bla*CTX-M genes in both the analyzed soils. The presence of β-lactamases in soil environments is not a rare phenomenon and has already been demonstrated, although not quantified, in previous studies (Allen et al., 2009; Lu et al., 2010; Chikwendu et al., 2011; Hartman et al., 2012; Walsh and Duffy, 2013; Jones-Dias et al., 2015; Li et al., 2015; Perron et al., 2015). Additionally, several studies have detected ESBL genes in a wide range of taxonomic groups, such as *Enterobacteriacae*, *Pseudomonas*, *Campylobacter*, *Dialister*, *Fusobacterium*, and *Provotella* in nosocomial environments (Shacheragi et al., 2010; Rocas and Siqueiras, 2013), *Sphingomonas* and *Sphingobium* in drinking and river water (Vaz-Moreira et al., 2011), and *Nocardia* and *Streptomyces* in soils (Ogawara et al., 1978; Khan et al., 2001).

Interestingly, the indicator species analyses (Supplementary Tables S4 and S5) of the microbiome data reveal that specific OTUs that were significantly more profuse in the antibiotic-amended microcosms often belonged to the same families as OTUs that were more abundant in the non-amended samples (Supplementary Tables S4 and S5); suggesting that ceftriaxone resistance was not specifically linked to distinct bacteria phyla. This, coupled to the fact that ESBLs are broadly distributed among microbial taxa and that they are often harbored on mobile genetic elements may suggest that their dissemination in soil is driven by horizontal gene transfer. Soil bacteria seemingly harbor β-lactamase enzymes as a protective mechanism against fungal β-lactam producers, and therefore, their acquisition may confer a selective advantage in soils containing β-lactam producing fungi. This idea is supported by Smillie et al. (2011), who found that horizontal gene transfer is shaped principally by ecology rather than geography or bacterial phylogeny. Nonetheless, the expanded capacity of these soils to mitigate the effects of ceftriaxone, a third generation cephalosporin not typically found in native soils, is not completely clear and a synergistic effect of ESBL activity together with other AR mechanisms such as eﬄux pumps cannot be dismissed.

The rapid decline in *E. coli* abundance in the unsterilized ceftriaxone-amended and the non-amended microcosms (**Figure 5**) supports the slurry data suggesting significant presence of *in-terra* β-lactamase activity; and indicates that the soils in the microcosms were characterized by high microbial diversity; In this context Van Elsas et al. (2012), indicate that the resistance of soils to invasion by alien species is directly associated with the diversity of the soil microbiome. The lack of native β-lactamase activity in the ceftriaxone-amended sterilized soils (three cycles of sterilization at 120°C), as observed by the constant *E. coli* abundance, undoubtedly can be explained by denaturation of native soil β-lactamases due to the autoclave treatment which generates a temperature almost twofold higher than the highest denaturation temperature (*T_m_*) reported to date for a β-lactamase [variant of *bla*TEM-1 with a *T_m_* of 69°C (Kather et al., 2008)]. It could be argued that the half life of ceftriaxone and other cephalosporin-like antibiotics is variable, depending on the experimental conditions (De Diego et al., 2010; Jiang et al., 2010; Braschi et al., 2013; Mitchell et al., 2013); however, it is never lower than 2 days and therefore we assumed that amendment of ceftriaxone every other day was sufficient to keep constant antibiotic activities in the soils.

The resistance of the soil microbial community to third generation cephalosporins, and the indication that this resistance is associated with ESBL activity, has huge implications for the development of AR in clinical environments because ESBLs are often associated with mobile gene elements such as transposons and insertion sequences, plasmids, integrons and integrative conjugative elements (Van Elsas and Bailey, 2002); thus, horizontal gene transfer of these elements may increase the spread of ARGs in pathogenic and opportunistic bacteria in the clinical environment. The potential horizontal transfer of ESBLs from soil bacteria to human pathogens is supported by the recent isolation of a carbapenem-resistant *Serratia marcescens* strain from a hospital in Japan that harbored a novel metallo- β-lactamase termed SMB-1 associated with a class 1 integron (Wachino et al., 2011). This enzyme was not closely related to any clinically-characterized β-lactamases, but instead was highly homologous to a metallo-β-lactamase termed AMO1, identified in the functional metagenomic analysis of an apple orchard soil (Donato et al., 2010).

## Conclusion

Our data demonstrates that exposure to cephalosporin at clinical doses does not significantly affect the bacterial community structure and microbial activity of the undisturbed analyzed soils. The AR phenomenon was strongly associated to biological activity and even more, due to β-lactamase activity associated with ESBL genes. This phenomenon has unknown clinical implications and therefore more studies are necessary to determinate the full scope of cephalosporin resistance mechanisms in undisturbed soils and elucidate their link to emerging clinical pathogens in the future.

## Conflict of Interest Statement

The authors declare that the research was conducted in the absence of any commercial or financial relationships that could be construed as a potential conflict of interest.

## References

[B1] Allen H. Moe L. Rodbumrer J. Gaarder A. Handelsman J. (2009). Functional metagenomics reveals diverse β-lactamases in a remote Alaskan soil. *ISME J.* 3 243–251. 10.1038/ismej.2008.86 18843302

[B2] Anderson M. Ellingsen K. McArdle B. (2006). Multivariate dispersion as a measure of beta diversity. *Ecol. Lett.* 9 683–693. 10.1111/j.1461-0248.2006.00926.x 16706913

[B3] Barnes A. Hastings T. Amyes S. (1994). Amoxicillin resistance in Scottish isolates of *Aeromonas salmonicida*. *J. Fish. Dis.* 17 357–363. 10.1111/j.1365-2761.1994.tb00231.x

[B4] Bert F. Branger C. Lambert-Zechovsky N. (2002). Identification of PSE and OXA β-lactamase genes in *Pseudomonas aeruginosa* using PCR-restriction fragment length polymorphism. *JAC* 50 11–18. 12096001 10.1093/jac/dkf069

[B5] Bhullar K. Waglechner N. Pawlowski A. Koteva A. Banks E. Johnston M. (2012). Antibiotic resistance is prevalent in an isolated cave microbiome. *PLoS ONE* 7:e34953 10.1371/journal.pone.0034953 PMC3324550 22509370

[B6] Binh C. Heuer H. Kaupenjohann M. Smalla K. (2008). Piggery manure used for soil fertilization is a reservoir for transferable antibiotic resistance plasmids. *FEMS Microb. Ecol.* 66 25–37. 10.1111/j.1574-6941.2008.00526.x 18557938

[B7] Braschi I. Blasioli S. Fellet C. Lorenzini R. Garelli A. Pori M. (2013). Persistence and degradation of new β-lactam antibiotics in the soil and water environment. *Chemosphere* 93 152–159. 10.1016/j.chemosphere.2013.05.016 23777677

[B8] Brusetti L. Glad T. Borin S. Myren P. Rizzi A. Johnsen P. (2008). Low prevalence of bla TEM genes in Arctic environments and agricultural soil and rhizosphere. *Microb. Ecol. Health D* 20 27–36. 10.1080/08910600701838244

[B9] Caporaso J. Bittinger K. Bushman F. DeSantis T. Andersen G. (2010a). PyNAST: a flexible tool for aligning sequences to a template alignment. *Bioinformatics* 26 266–267. 10.1093/bioinformatics/btp636 19914921 PMC2804299

[B10] Caporaso J. Kuczynski J. Stombaugh J. Bittinger K. Bushman F. Costello E. (2010b). QIIME allows analysis of high-throughput community sequencing data. *Nat. Methods* 7 335–336. 10.1038/nmeth.f.303 20383131 PMC3156573

[B11] Casida L. Klein D. Santoro T. (1964). Soil dehydrogenase activity. *Soil. Sci.* 98 371–376. 10.1097/00010694-196412000-00004

[B12] Chikwendu C. Ibe S. Okpokwasili G. (2011). Detection of blaSHV and blaTEM β-lactamase genes in multi-resistant *Pseudomonas* isolates from environmental sources. *Afr. J. Microbiol. Res.* 5 2067–2074. 10.5897/AJMR11.149

[B13] Davies J. Davies D. (2010). Origins and evolution of antibiotic resistance. *Microbiol. Mol. Biol. Rev.* 74 417–433. 10.1128/MMBR.00016-10 20805405 PMC2937522

[B14] D’Costa V. King C. Kalan L. Morar M. Sung W. Schwarz C. (2011). Antibiotic resistance is ancient. *Nature* 477 457–461. 10.1038/nature10388 21881561

[B15] De Diego M. Godoy G. Mennickent S. (2010). Chemical stability of ceftriaxone by a validated stability-indicating liquid chromatographic method. *J. Chil. Chem. Soc.* 3 1–3.

[B16] Dhillon R. Clark J. (2012). ESBLs: a clear and present danger? *Crit. Care Res. Pract.* 12 1–11. 10.1155/2012/625170 PMC3135063 21766013

[B17] Dimitriu P. Grayston S. (2010). Relationship between soil properties and patterns of bacterial β-diversity across reclaimed and natural boreal forest soils. *Microb. Ecol.* 59 563–573. 10.1007/s00248-009-9590-0 19830478

[B18] Donato J. Moe L. Converse E. Smart K. Berklein F. McManus P. (2010). Metagenomic analysis of apple orchard soil reveals antibiotic resistance genes encoding predicted bifunctional proteins. *Appl. Environ. Microbiol.* 76 4396–4401. 10.1128/AEM.01763-09 20453147 PMC2897439

[B19] Dowd S. Callaway T. Wolcott R. Sun Y. McKeehan T. Hagevoort R. (2008a). Evaluation of the bacterial diversity in the feces of cattle using 16rDNA bacterial tag-encoded FLX amplicon pyrosequencing (bTEFAP). *BMC Microbiol.* 8:125 10.1186/1471-2180-8-125 PMC2515157 18652685

[B20] Dowd S. Sun Y. Wolcott R. Domingo A. Carrol J. (2008b). Bacterial tag-encoded FLX amplicon pyrosequencing (bTEFAP) for microbiome studies: bacterial diversity in the ileum of newly weaned *Salmonella*-infect pigs. *Foodborne Pathog. Dis.* 5 479–552. 10.1089/fpd.2008.0107 18713063

[B21] Edgar R. (2010). Search and clustering orders of magnitude faster than BLAST. *Bioinformatics* 26 2460–2461. 10.1093/bioinformatics/btq461 20709691

[B22] Falagas M. Karageorgopoulos D. (2009). Extended-spectrum β-lactamase-producing organisms. *J. Hosp. Infect.* 73 345–354. 10.1016/j.jhin.2009.02.021 19596491

[B23] Forsberg K. Patel S. Gibson M. Lauber C. Knight R. Fierer N. (2014). Bacterial phylogeny structures soil resistomes across habitats. *Nat. Lett.* 509 612–616. 10.1038/nature13377 PMC4079543 24847883

[B24] Foucault C. Brouqui P. (2007). How to fight antimicrobial resistance. *FEMS Immunol. Med. Microbiol.* 49 173–183. 10.1111/j.1574-695X.2006.00172.x 17181560

[B25] Haas B. Gevers D. Earl M. Feldgarden M. Ward D. (2011). Chimeric 16S rRNA sequence information and detection in Sanger and 454-pyrosequenced PCR amplicons. *Genome Res.* 21 494–504. 10.1101/gr.112730.110 21212162 PMC3044863

[B26] Hartman A. Locatelli A. Amoureux A. Depret G. Jolivet C. Gueneau E. (2012). Occurrence of CTX-M producing *Escherichia coli* in soils, cattle and farm environment in France (Burgundy region). *Front. Microbiol.* 3:83 10.3389/fmicb.2012.00083 PMC3297819 22408639

[B27] Jiang M. Wang L. Ji R. (2010). Biotic and abiotic degradation of four cephalosporin antibiotics in a lake surface water and sediment. *Chemosphere* 80 1399–1405. 10.1016/j.chemosphere.2010.05.048 20579689

[B28] Jones R. Robeson M. Lauber C. Hamady M. Knight R. Fierer N. (2009). A comprehensive survey of soil acidobacterial diversity using pyrosequencing and clone library analyses. *ISME J.* 3 442–453. 10.1038/ismej.2008.127 19129864 PMC2997719

[B29] Jones-Dias D. Manageiro V. Canica M. (2015). Influence of agricultural practice on mobile bla genes: incI1-bearing CTX-M, SHV, CMY and TEM in *Escherichia coli* from intensive farming soils. *Environ. Microbiol.* 10.1111/1462-2920.13021 [Epub ahead of print]. 26279315

[B30] Kather I. Jakob P. Dobbek H. Schmid X. (2008). Increased folding stability of TEM-1 β-lactamase by in vitro selection. *J. Mol. Biol.* 383 238–251. 10.1016/j.jmb.2008.07.082 18706424

[B31] Khan Z. Al-Sayer H. Chugh T. Chandy R. Provost F. Boiron P. (2001). Antimicrobial susceptibility profile of soil isolates of Nocardia asteroids from Kuwait. *ECCMID* 6 94–98. 10.1046/j.1469-0691.2000.00026.x 11168079

[B32] Kim M. Boldgiv B. Singh D. Chun J. Lkhagva A. Adams J. (2012). Structure of soil bacterial communities in relation to environmental variables in a semi-arid region of Mongolia. *J. Arid Environ.* 89 38–44. 10.1016/j.jaridenv.2012.09.014

[B33] Knapp W. Dolfing J. Ehlert I. Graham W. (2010). Evidence of increasing 429 antibiotic resistance gene abundances in archived soils since 1940. *Environ. Sci. Technol.* 44 580–587. 10.1021/es901221x 20025282

[B34] Kummerer K. (2009). Antibiotics in the aquatic environment- a review- part I. *Chemosphere* 75 417–434. 10.1016/j.chemosphere.2008.11.086 19185900

[B35] Kummerer K. Henninger A. (2003). Promoting resistance by the emission of antibiotics from hospitals and households into eﬄuent. *Clin. Microbiol. Infec.* 9 1203–1214. 10.1111/j.1469-0691.2003.00739.x 14686985

[B36] Li B. Yang Y. Ma L. Ju F. Guo F. Tiedje J. (2015). Metagenomic and network analysis reveal wide distribution and co-ocurrence of environmental antibiotic resistance genes. *ISME J.* 9 2490–2502. 10.1038/ismej.2015.59 25918831 PMC4611512

[B37] Lozupone C. Knight R. (2005). Unifrac: a new phylogenetic method for comparing microbial communities. *Appl. Environ. Microbiol.* 71 8228–8235. 10.1128/AEM.71.12.8228-8235.2005 16332807 PMC1317376

[B38] Lu S. Ya-Li Z. Sui-Na G. Tian-Yu L. Zhuo-Ming Y. Dong-Sheng Z. (2010). High diversity of extended-spectrum β-lactamase-producing bacteria in an urban river sediment habitat. *Appl. Environ. Microbiol.* 76 5972–5976. 10.1128/AEM.00711-10 20639374 PMC2935080

[B39] Mazzariol A. Mammina C. Koncan R. Di Gaetano V. Di Carlo P. Cipolla D. (2011). A novel VIM-type metallo-β-lactamase (VIM-14) in a *Pseudomonas aeruginosa* clinical isolate from a neonatal intensive care unit. *Clin. Microbiol. Infec.* 17 722–724. 10.1111/j.1469-0691.2010.03424.x 21521413

[B40] McHugh T. Koch G. Schwartz E. (2014). Minor changes in soil bacteria and fungal community composition occur in response to monsoon precipitation in a semiarid grassland. *Microb. Ecol.* 68 370–378. 10.1007/s00248-014-0416-3 24743883

[B41] McLain J. Williams C. (2010). “Development of antibiotic resistance in bacteria of soils irrigated with reclaimed wastewater,” in *Proceedings of 5th National Decennial Irrigation Conference*, Phoenix Convention Center, Phoenix, AZ.

[B42] Mitchell S. Ullman J. Teel A. Watts R. (2013). pH and temperature effects on the hydrolysis of three β-lactam antibiotics: ampicillin, cefalotin and cefoxitin. *Sci. Total Environ.* 46 547–555. 10.1016/j.scitotenv.2013.06.027 23948499

[B43] Monstein H. Ostholm-Balkhed A. Nilsson M. Nilsson M. Dornbusch K. Nilsson L. (2007). multiplex PCR amplification assay for the detection of blaSHV, blaTEM and blaCTX-M genes in *Enterobacteriaceae*. *APMIS* 115 1400–1408. 10.1111/j.1600-0463.2007.00722.x 18184411

[B44] Munir M. Wong K. Xagoraraki I. (2011). Release of antibiotic resistant bacteria and genes in the eﬄuent of biosolids of five wastewater utilities in Michigan. *Water. Res.* 45 681–693. 10.1016/j.watres.2010.08.033 20850863

[B45] Negreanu Y. Pasternak Z. Jurkevitch E. Cytryn E. (2012). Impact of treated wastewater irrigation on antibiotic resistance in agricultural soils. *Environ. Sci. Technol.* 46 4800–4808. 10.1021/es204665b 22494147

[B46] Nordmann P. Poirel L. Carrer A. Toleman A. Walsh T. (2011). How to detect NDM-1 producers. *J. Clin. Microbiol.* 49 718–721. 10.1128/JCM.01773-10 21123531 PMC3043507

[B47] Ogawara H. Horikawa S. Shimada-Miyoshi S. Yasuzawa K. (1978). Production and property of B-Lactamases in *Streptomyces*: comparison of the strains isolated newly and thirty years ago. *Antimicrob. Agents Chemother.* 13 865–870. 10.1128/AAC.13.5.865 666306 PMC352346

[B48] Pasternak Z. Al-Ashhab A. Gatica J. Gafny R. Avraham S. Minz D. (2013). Spatial and temporal biogeography of soil microbial communities in arid and semiarid regions. *PLoS ONE* 8:e69705 10.1371/journal.pone.0069705 PMC3724898 23922779

[B49] Paterson D. Hujer K. Hujer A. Yeiser B. Bonomo M. Rice L. (2003). Extended-spectrum β-lactamases in *Klebsiella pneumoniae* bloodstream isolates from seven countries: dominance and widespread prevalence of SHV- and CTX-M- type β-lactamases. *Antimicrob. Agents Chemother.* 47 3554–3560. 10.1128/AAC.47.11.3554-3560.2003 14576117 PMC253771

[B50] Perez F. Endimiani A. Hujer H. Bonomo R. (2007). The continuing challenge of ESBLs. *Curr. Opin. Pharmacol.* 7 459–469. 10.1016/j.coph.2007.08.003 17875405 PMC2235939

[B51] Perron G. Whyte L. Turnbaugh P. Goordial J. Hanage W. Dantas G. (2015). Functional characterization of bacteria isolated from ancient artic soil exposes diverse resistance mechanisms to modern antibiotics. *PLoS ONE* 10:e0069533 10.1371/journal.pone.0069533 PMC4373940 25807523

[B52] Pitout J. (2006). Molecular detection of bacteria producing newer types of β-lactamases. *Curr. Genomics* 7 171–177. 10.2174/138920206777780238

[B53] Pitout J. Hossain A. Nancy H. (2004). Phenotypic and molecular detection of CTX-M- β-lactamases produced by *Eschericha coli* and *Klebsiella* spp. *J. Clin. Microbiol.* 42 5715–5721. 10.1128/JCM.42.12.5715-5721.2004 15583304 PMC535227

[B54] Rampelotto P. Ferreira A. Barboza A. Roesh L. (2013). Changes in diversity, abundance, and structure of soil bacterial communities in Brazilian savanna under different land use systems. *Microb. Ecol.* 66 593–607. 10.1007/s00248-013-0235-y 23624541

[B55] Robin F. Delmas J. Chanal C. Sirot D. Sirot J. Bonnet R. (2005). TEM-109 (CMT-5), a natural complex mutant of TEM-1 β- Lactamase combining the amino acid substitutions of TEM-6 and TEM-33 (IRT-5). *Antimicrob. Agents Chemother.* 49 4443–4447. 10.1128/AAC.49.11.4443-4447.2005 16251281 PMC1280126

[B56] Rocas I. Siqueiras J. (2013). Antibiotic resistance genes in anaerobic bacteria isolated from primary dental root canal infections. *Anaerobe* 18 576–580. 10.1016/j.anaerobe.2012.10.001 23108290

[B57] Schurer J. Rosswall T. (1982). Fluorescein diacetate hydrolysis as a measure of total microbial activity in soil and litter. *Appl. Environ. Microbiol.* 43 256–1261. 10.1128/aem.43.6.1256-1261.1982 PMC244223 16346026

[B58] Shacheragi F. Shakibaie M. Noveiri H. (2010). Molecular identification of ESBL genes aeruginosa strains isolated from burn patients by PCR, RFLP and sequencing techniques. *Int. J. Biol. Life Sci.* 6 138–142.

[B59] Smalla K. Heuer H. Gotz A. Niemeyer D. Krogerrecklenfort E. Tietze E. (2000). Exogenous isolation of antibiotic resistance plasmids from piggery manure slurries reveals a high prevalence and diversity of IncQ-like plasmids. *Appl. Environ. Microbiol.* 66 4854–4862. 10.1128/AEM.66.11.4854-4862.2000 11055935 PMC92391

[B60] Smillie C. Smith M. Friedman J. Cordero O. David L. Alm E. (2011). Ecology drives a global network of gene exchange connecting the human microbiome. *Nature* 480 241–244. 10.1038/nature10571 22037308

[B61] Van Elsas J. Bailey M. (2002). The ecology of transfer of mobile genetic elements. *FEMS Microbiol. Ecol.* 42 187–197. 10.1111/j.1574-6941.2002.tb01008.x 19709278

[B62] Van Elsas J. Chiurazzi M. Mallon C. Elhottova D. Kristufek V. Salles F. (2012). Microbial diversity determines the invasion of soil by a bacterial pathogen. *Proc. Natl. Acad. Sci. U.S.A.* 109 1159–1164. 10.1073/pnas.1109326109 22232669 PMC3268289

[B63] Vaz-Moreira I. Nunes O. Manaia C. (2011). Diversity and antibiotic resistance patterns of Sphingomonadaceae isolates from drinking water. *Appl. Environ. Microbiol.* 77 5697–5706. 10.1128/AEM.00579-11 21705522 PMC3165245

[B64] Wachino J. Yoshida H. Yamane K. Suzuki S. Matsui M. Yamagishi T. (2011). SMB-1, a novel subclass B3 metallo-β-lactamase, associated with ISCR1 and class 1 integron, from a carbapenem-resistant *Serratia marcescens* clinical isolate. *Antimicrob. Agents Chemother.* 55 5143–5149. 10.1128/AAC.05045-11 21876060 PMC3195065

[B65] Walsh F. Duffy B. (2013). The culturable soil antibiotic resistome: a community of multidrug resistant bacteria. *PLoS ONE* 8:e65567 10.1371/journal.pone.0065567 PMC3680443 23776501

[B66] Watanabe K. Tanaka A. Imase K. Tokunaga K. Sugano H. Kai A. (2005). Short-term effects of amoxicillin on bacterial communities in manured soil. *FEMS Microbiol. Ecol.* 62 290–302. 10.1111/j.1574-6941.2007.00393.x 17991020

[B67] Yang H. Byelashov O. Geornaras I. Goodridge L. Nightingale K. Belk K. (2010). Presence of antibiotic-resistant commensal bacteria in samples from agriculture, city and national park environments evaluated by sstandard culture and real-time PCR methods. *Can. J. Microbiol.* 56 761–770. 10.1139/w10-060 20921986

