# Supplementary material for: Resistance of Undisturbed Soil Microbiomes to Ceftriaxone Indicates Extended Spectrum β-Lactamase Activity
Source: Front Microbiol. 2015 Nov 10;6:1233. doi: 10.3389/fmicb.2015.01233 (PMC4639628; doi:10.3389/fmicb.2015.01233)
Supplement: Supplementary file 1 [file Presentation_1.PDF]

Table S1. Statistical differences in phyla between treatments for each soil analyzed according to taxonomic assignment of OTUs. Significant differences evaluated using Kruskall-Wallis test at confidence level of 95%.

		Phylum									
		Acidobacteria	Actinobacteria	Chloroflexi	Cyanobacteria	Firmicutes	Gemmatimonadetes	Planctomycetes	Proteobacteria	Verrucomicrobia	Others
Differences between groups (p values*)	sandy loam soil	>0.05	0.012	>0.05	>0.05	0.042	>0.05	>0.05	>0.05	>0.05	>0.05
	clay soil	>0.05	>0.05	>0.05	>0.05	>0.05	>0.05	>0.05	>0.05	>0.05	>0.05

* Indicate differences between all the treatments (T0, T7C, T7A, T14C and T14A) for a specific phylum

Abbreviations: T0- experimental initiation; T7- seven days after experimental initiation; T14- 14 days after experimental initiation; C- Non-amended control microcosms; A- antibiotic-amended microcosms.

Table S2. Statistical differences in diversity indexes between treatments for each soil analyzed. T0 correspond to time zero, T7 and T14 correspond to time 7 and 14 days after the beginning of the experiment; C indicates control treatment and A indicates antibiotic treatment. Significant differences evaluated using Kruskall-Wallis test at confidence level of 95%.

		Taxa_S	Individual	Dominance	Simpson	Shannon	Evenness	Chao-1
**sandy loam soil**	Differences between groups* (p value)	0.018	>0.05	>0.05	>0.05	>0.05	>0.05	0.023
	Differences between treatments** (p value)	T0C vs T14C (p= 0.008)<br>T0C vs T14A (p= 0.018)<br>T7A vs T14C (p= 0.014)<br>T7A vs T14A (p= 0.028)	---	---	---	---	---	T0C vs T14C (p= 0.018)<br>T0C vs T14A (p= 0.014)<br>T7A vs T14C (p= 0.022)<br>T7A vs T14A (p= 0.018)
**clay soil**	Differences between groups* (p value)	>0.05	>0.05	>0.05	>0.05	0.049	>0.05	>0.05
	Differences between treatments** (p value)	---	---	---	---	T0C vs T7C (p= 0.011)<br>T7C vs T14C (p= 0.028)<br>T7C vs T14A (p= 0.028)	---	---

Abbreviations: T0- experimental initiation; T7- seven days after experimental initiation; T14- 14 days after experimental initiation; C- Non-amended control microcosms; A- antibiotic-amended microcosms.

* Indicate differences between all the treatments (T0, T7C, T7A, T14C and T14A) for a specific phylum

** Indicate which comparisons were statistically different among the treatments.

Table S3. Significance tests for β-diversity among bacterial community profiles by PERMDISP method using unweighted and weighted UniFrac on QIIME. A) sandy loam soil. B) clay soil. Pairwise comparisons: observed p-value below diagonal, permuted (999 permutations) p-value above diagonal. T7 and T14 correspond to time 7 and 14 days after the beginning of the experiment; C indicates control treatment and A indicates antibiotic treatment.

**A**	**sandy loam soil**										
**Unweighted UniFrac**						**Weighted UniFrac**					
	T14A	T7A	T0C	T14C	T7C		T14A	T7A	T0C	T14C	T7C
T14A		0.190	0.075	0.915	0.257	T14A		0.286	0.122	0.598	0.774
T7A	0.174		0.343	0.050	0.556	T7A	0.278		0.186	0.882	0.186
T0C	0.050	0.330		0.003	0.106	T0C	0.124	0.196		0.441	0.09
T14C	0.925	0.044	0.001		0.083	T14C	0.593	0.886	0.447		0.694
T7C	0.281	0.564	0.092	0.073		T7C	0.781	0.179	0.089	0.683	

**B**	**clay soil**										
**Unweighted UniFrac**						**Weighted UniFrac**					
	T14A	T7A	T0C	T14C	T7C		T14A	T7A	T0C	T14C	T7C
T14A		0.859	0.429	0.606	0.156	T14A		0.853	0.862	0.647	0.392
T7A	0.856		0.900	0.815	0.362	T7A	0.840		0.969	0.456	0.186
T0C	0.399	0.899		0.816	0.212	T0C	0.824	0.963		0.374	0.191
T14C	0.609	0.804	0.822		0.449	T14C	0.613	0.400	0.334		0.386
T7C	0.15	0.33	0.203	0.449		T7C	0.363	0.207	0.194	0.350	

Table S4. Indicator OTUs from the sandy loam soil showing significant differences in relative abundance in response to antibiotic amendment, determined using the Monte Carlo test of significance at 15999 permutations. The phylogeny is based on results obtained in QIIME.

OTU	Treatment	p	Phylum	Class	Order	Family	Genus
OTU2557	T0C	0.0218	Acidobacteria	Acidobacteria-6	iii1-15	mb2424	---
OTU1451	T0C	0.0444	Acidobacteria	Acidobacteria-6	iii1-15	---	---
OTU1624	T0C	0.0091	Acidobacteria	Chloracidobacteria	---	---	---
OTU2128	T0C	0.0239	Acidobacteria	Chloracidobacteria	---	---	---
OTU2031	T0C	0.0241	Acidobacteria	Chloracidobacteria	---	---	---
OTU495	T0C	0.0268	Acidobacteria	Chloracidobacteria	---	---	---
OTU1260	T0C	0.0376	Acidobacteria	Chloracidobacteria	---	---	---
OTU2144	T0C	0.0391	Acidobacteria	iii1-8	DS-18	---	---
OTU405	T0C	0.0356	Acidobacteria	Sva0725	Sva0725	---	---
OTU2491	T0C	0.0348	Actinobacteria	Actinobacteria	Actinomycetales	Actinosynnemataceae	Saccharothrix
OTU603	T0C	0.0456	Actinobacteria	Actinobacteria	Actinomycetales	Actinosynnemataceae	Saccharothrix
OTU1363	T0C	0.0259	Actinobacteria	Actinobacteria	Actinomycetales	Geodermatophilaceae	---
OTU681	T0C	0.0133	Actinobacteria	Actinobacteria	Actinomycetales	Microbacteriaceae	---
OTU596	T0C	0.0248	Actinobacteria	Actinobacteria	Actinomycetales	Microbacteriaceae	---
OTU412	T0C	0.0083	Actinobacteria	Actinobacteria	Actinomycetales	Micrococcaceae	---
OTU1928	T0C	0.0133	Actinobacteria	Actinobacteria	Actinomycetales	Micrococcaceae	---
OTU732	T0C	0.0301	Actinobacteria	Actinobacteria	Actinomycetales	Micrococcaceae	---
OTU1412	T0C	0.0358	Actinobacteria	Actinobacteria	Actinomycetales	Micrococcaceae	---
OTU318	T0C	0.0438	Actinobacteria	Actinobacteria	Actinomycetales	Micromonosporaceae	---
OTU1691	T0C	0.008	Actinobacteria	Actinobacteria	Actinomycetales	Mycobacteriaceae	Mycobacterium
OTU297	T0C	0.0236	Actinobacteria	Actinobacteria	Actinomycetales	Mycobacteriaceae	Mycobacterium
OTU2552	T0C	0.0126	Actinobacteria	Actinobacteria	Actinomycetales	Nocardioidaceae	---
OTU104	T0C	0.0334	Actinobacteria	Actinobacteria	Actinomycetales	Nocardioidaceae	---
OTU230	T0C	0.0349	Actinobacteria	Actinobacteria	Actinomycetales	Nocardioidaceae	Kribbella
OTU961	T0C	0.0489	Actinobacteria	Actinobacteria	Actinomycetales	Streptomycetaceae	Streptomyces
OTU313	T0C	0.0333	Actinobacteria	Actinobacteria	Actinomycetales	Streptosporangiaceae	---
OTU1206	T0C	0.0116	Actinobacteria	Actinobacteria	Actinomycetales	---	---
OTU262	T0C	0.0133	Actinobacteria	Actinobacteria	Actinomycetales	---	---
OTU939	T0C	0.0234	Actinobacteria	Actinobacteria	Actinomycetales	---	---
OTU1091	T0C	0.0133	Actinobacteria	MB-A2-108	0319-7L14	---	---
OTU1476	T0C	0.0239	Actinobacteria	MB-A2-108	0319-7L14	---	---
OTU2413	T0C	0.0463	Actinobacteria	Nitriliruptoria	Euzebyales	Euzebyaceae	Euzebya
OTU2357	T0C	0.0133	Actinobacteria	Rubrobacteria	Rubrobacterales	Rubrobacteraceae	Rubrobacter
OTU2029	T0C	0.0261	Actinobacteria	Rubrobacteria	Rubrobacterales	Rubrobacteraceae	Rubrobacter
OTU1012	T0C	0.0133	Actinobacteria	Thermoleophilia	Gaiellales	Gaiellaceae	---
OTU2314	T0C	0.0344	Actinobacteria	Thermoleophilia	Solirubrobacterales	---	---
OTU2084	T0C	0.0133	Chloroflexi	Thermomicrobia	JG30-KF-CM45	---	---
OTU534	T0C	0.0133	Cyanobacteria	Chloroplast	Streptophyta	---	---
OTU2185	T0C	0.0132	Fibrobacteres	Fibrobacteria	258ds10	---	---
OTU32	T0C	0.0088	Proteobacteria	Alphaproteobacteria	Rhizobiales	Hyphomicrobiaceae	Devosia;s
OTU1309	T0C	0.0133	Proteobacteria	Alphaproteobacteria	Rhizobiales	Hyphomicrobiaceae	---

OTU430	T0C	0.0133	Proteobacteria	Alphaproteobacteria	Rhizobiales		---
OTU455	T0C	0.0096	Proteobacteria	Alphaproteobacteria	Rhodospirillales		---
OTU2466	T0C	0.0235	Proteobacteria	Alphaproteobacteria	Rhodospirillales	Rhodospirillaceae	---
OTU1062	T0C	0.0428	Proteobacteria	Alphaproteobacteria	Sphingomonadales	Sphingomonadaceae	---
OTU1469	T0C	0.0245	Proteobacteria	Alphaproteobacteria	---	---	---
OTU199	T0C	0.0103	Proteobacteria	Betaproteobacteria	---	---	---
OTU2191	T0C	0.0132	Proteobacteria	Betaproteobacteria	---	---	---
OTU1796	T0C	0.0412	Proteobacteria	Deltaproteobacteria	Myxococcales	Polyangiaceae	---
OTU1028	T0C	0.0488	Proteobacteria	Deltaproteobacteria	Syntrophobacterales	Syntrophobacteraceae	---
OTU1876	T0C	0.0133	Proteobacteria	Gammaproteobacteria	HTCC2188	211ds20	---
OTU1258	T0C	0.0235	Verrucomicrobia	Opitutae	Opitutales	Opitutaceae	Opitutus

OTU1210	T7C	0.0104	Firmicutes	Bacilli	Bacillales	Bacillaceae	Bacillus
OTU1097	T7C	0.046	Firmicutes	Bacilli	Bacillales	Bacillaceae	Bacillus
OTU2532	T7C	0.0104	Firmicutes	Bacilli	Bacillales	Paenibacillaceae	Brevibacillus
OTU840	T7C	0.0403	Firmicutes	Clostridia	Clostridiales	Clostridiaceae	Caloramator
OTU901	T7C	0.0104	Firmicutes	Clostridia	Clostridiales	Clostridiaceae	Clostridium
OTU286	T7C	0.0104	Firmicutes	Clostridia	Clostridiales	Clostridiaceae	Clostridium
OTU157	T7C	0.0458	Firmicutes	Clostridia	Clostridiales	Clostridiaceae	Clostridium
OTU2132	T7C	0.0347	Firmicutes	Clostridia	Clostridiales	Clostridiaceae	---
OTU421	T7C	0.0104	Firmicutes	Clostridia	Clostridiales	Lachnospiraceae	---
OTU1925	T7C	0.0104	Firmicutes	Clostridia	Clostridiales	Peptococcaceae	---
OTU2595	T7C	0.0218	Firmicutes	Clostridia	Clostridiales	Peptococcaceae	---
OTU263	T7C	0.0205	Firmicutes	Clostridia	Clostridiales	Symbiobacteriaceae	Symbiobacterium
OTU1605	T7C	0.0104	Firmicutes	Clostridia	Clostridiales	---	---
OTU1743	T7C	0.0301	Proteobacteria	Betaproteobacteria	Burkholderiales	Oxalobacteraceae	---
OTU2685	T7C	0.0453	Proteobacteria	Betaproteobacteria	Burkholderiales	Oxalobacteraceae	---
OTU2249	T7A	0.0106	Actinobacteria	Actinobacteria	Actinomycetales	Geodermatophilaceae	---
OTU0	T7A	0.0307	Actinobacteria	Actinobacteria	Actinomycetales	Micromonosporaceae	---
OTU1870	T7A	0.0106	Proteobacteria	Alphaproteobacteria	Caulobacterales	Caulobacteraceae	Mycoplana
OTU2387	T14C	0.0434	Firmicutes	Clostridia	Clostridiales	Clostridiaceae	Clostridium
OTU2676	T14C	0.0316	Firmicutes	Clostridia	Clostridiales	Symbiobacteriaceae	Symbiobacterium
OTU861	T14C	0.0105	Firmicutes	---	---	---	---
OTU131	T14C	0.0318	Proteobacteria	Alphaproteobacteria	Rhodospirillales	Rhodospirillaceae	Azospirillum
OTU2543	T14C	0.0373	Proteobacteria	Alphaproteobacteria	Rhodospirillales	Rhodospirillaceae	Azospirillum
OTU2706	T14C	0.0329	Proteobacteria	Betaproteobacteria	Burkholderiales	Oxalobacteraceae	---
OTU1220	T14C	0.0206	Verrucomicrobia	Opitutae	Opitutales	Opitutaceae	Opitutus
OTU601	T14A	0.034	Proteobacteria	Alphaproteobacteria	Rhodospirillales	Rhodospirillaceae	---

Abbreviations: Non-amended control microcosms (C), antibiotic-amended microcosms (A); experimental initiation (T0), seven days after experimental initiation (T7), 14 days after experimental initiation (T14)

Table S5. Indicator OTUs from the clay soil showing significant differences in relative abundance in response to antibiotic amendment, determined using the Monte Carlo test of significance at 15999 permutations. The phylogeny is based on results obtained in QIIME.

OTU	Treatment	p value	Phylum	Class	Order	Family	Genus
OTU2316	T0C	0.0484	Acidobacteria	Acidobacteria	Acidobacteriales	Koribacteraceae	Candidatus Koribacter
OTU1242	T0C	0.0079	Actinobacteria	Actinobacteria	Actinomycetales	Micromonosporaceae	---
OTU1184	T0C	0.0163	Actinobacteria	Actinobacteria	Actinomycetales	Micromonosporaceae	---
OTU424	T0C	0.0333	Actinobacteria	Actinobacteria	Actinomycetales	---	---
OTU2052	T0C	0.0328	Proteobacteria	Alphaproteobacteria	Rhodospirillales	Rhodospirillaceae	---
OTU1249	T0C	0.0437	Proteobacteria	Deltaproteobacteria	---	---	---
OTU810	T0C	0.0324	Proteobacteria	Gammaproteobacteria	Xanthomonadales	Sinobacteraceae	---
OTU31	T7C	0.0433	Actinobacteria	Actinobacteria	Actinomycetales	Mycobacteriaceae	Mycobacterium
OTU1987	T7C	0.0419	Actinobacteria	Actinobacteria	Actinomycetales	---	---
OTU122	T7C	0.0315	Proteobacteria	Alphaproteobacteria	Caulobacterales	Caulobacteraceae	Phenylobacterium
OTU651	T7C	0.0199	Proteobacteria	Alphaproteobacteria	Ellin329	---	---
OTU106	T7C	0.0421	Proteobacteria	Alphaproteobacteria	Rhodobacterales	Hyphomonadaceae	---
OTU1018	T7C	0.0089	Proteobacteria	Alphaproteobacteria	Rhodospirillales	---	---
OTU1197	T7C	0.0293	Proteobacteria	Deltaproteobacteria	Myxococcales	---	---
OTU1046	T7C	0.0069	Proteobacteria	Gammaproteobacteria	Xanthomonadales	Sinobacteraceae	---
OTU1845	T7A	0.0379	Acidobacteria	Acidobacteria	Acidobacteriales	Koribacteraceae	---
OTU887	T7A	0.0471	Acidobacteria	Acidobacteria-5	---	---	---
OTU961	T7A	0.0395	Planctomycetes	Phycisphaerae	---	---	---
OTU1917	T7A	0.0413	Proteobacteria	Deltaproteobacteria	Syntrophobacterales	Syntrophobacteraceae	---
OTU381	T7A	0.0101	Proteobacteria	Gammaproteobacteria	Xanthomonadales	Sinobacteraceae	---
OTU51	T14C	0.0288	Acidobacteria	Acidobacteria	Acidobacteriales	Acidobacteriaceae	---
OTU1043	T14C	0.0313	Acidobacteria	Acidobacteria-5	---	---	---
OTU693	T14C	0.0311	Acidobacteria	Acidobacteria-6	iii1-15	---	---
OTU1676	T14C	0.024	Gemmatimonadetes	Gemmatimonadetes	---	---	---
OTU2318	T14C	0.0021	Proteobacteria	Alphaproteobacteria	Rhodospirillales	Acetobacteraceae	---
OTU105	T14A	0.0274	Acidobacteria	Acidobacteria	Acidobacteriales	Koribacteraceae	---
OTU643	T14A	0.0302	Acidobacteria	Acidobacteria-6	CCU21	---	---
OTU502	T14A	0.034	Actinobacteria	Actinobacteria	Actinomycetales	Nocardiaceae	Nocardia
OTU2023	T14A	0.0446	Proteobacteria	Betaproteobacteria	Burkholderiales	Burkholderiaceae	Burkholderia

Abbreviations: Non-amended control microcosms (C), antibiotic-amended microcosms (A); experimental initiation (T0), seven days after experimental initiation (T7), 14 days after experimental initiation (T14).

Table S6. Primers used for amplification of ESBL genes.

Target	Primer	Sequence (5' to 3')	Program	Product size (bp)	References
blaOXA	OXA-10F<br>OXA-10R	TCAACAAATCGCCAGAAG<br>TCCCACACCAGAAAAACCA	*Touchdown PCR1: initial denaturation of 94°C for 180 s, 20 initial cycles of 94°C for 30 s, annealing from 60°C to 50°C for 30 s (diminution of 5°C per cycle) and elongation of 72°C for 60 s, followed by 15 cycles with denaturation of 94°C for 30 s, annealing of 50°C for 30 s and elongation of 72°C for 60 s with a final elongation of 72°C for 300 s.	277	Bert et al. (2002)
blaSHV	bla-SHV.SE<br>bla-SHV.AS	ATGCGTTATATTCGCCTGTG<br>TGCTTTGTTATTCGGGCCAA		747	Paterson et al. (2003)
blaTEM	TEM-164.SE<br>TEM-164.AS	TCGCCGCATACACTATTCTCAGAATGA<br>ACGCTCACCGGCTCCAGATTTAT	*Touchdown PCR2: initial denaturation of 94°C for 180 s, 20 initial cycles of 94°C for 30 s, annealing from 65°C to 55°C for 30 s (diminution of 5°C per cycle) and elongation of 72°C for 60 s, followed by 10 cycles with denaturation of 94°C for 30 s, annealing of 55°C for 30 s and elongation of 72°C for 60 s with a final elongation of 72°C for 300 s.	445	Monstein et al. (2007)
blaVIM	VIM F<br>VIM R	CAGATTGCCGATGGTGTTTGG<br>AGGTGGGCCATTCAGCCAGA		523	Mazzaroli et al. (2011)
blaCTX-M	CTXM1-F3<br>CTXM1-R2	GACGATGTCACTGGCTGAGC<br>AGCCGCCGACGCTAATACA	Initial denaturation of 94°C for 300 s followed by 30 cycles of 94°C for 30 s, annealing from 68°C for 30 s and elongation of 72°C for 60 s, and a final elongation of 72°C for 600 s.	499	Pitout et al. (2004)
blaNDM	NDM-Fm<br>NDM-RM	GGTTTGGCGATCTGGTTTTC<br>CGGAATGGCTCATCACGATC	Initial denaturation of 94°C for 300 s, 30 cycles of 94°C for 30 s, annealing of 65°C for 30 s and elongation of 72°C for 60 s, followed by a final elongation step of 72°C for 600 s.	621	Nordmann et al. (2011)

*Touchdown PCR1 was used to target genes blaOXA and blaSHV and touchdown PCR2 was used to target genes blaTEM and blaVIM.

Bert F., Branger C., Lambert-Zechovsky N. 2002. Identification of PSE and OXA β-lactamase genes in *Pseudomonas aeruginosa* using PCR-restriction fragment length polymorphism. JAC 50:11-18.

Paterson D., Hujer K., Hujer A., Yeiser B., Bonomo M., Rice L., Bonomo R., International Klebsiella study group. 2003. Extended-spectrum β-lactamases in *Klebsiella pneumoniae* bloodstream isolates from seven countries: Dominance and widespread prevalence of SHV- and CTX-M- type β-lactamases. Antimicrob. Agents Chemother. 47:3554-3560.

Monstein H., Ostholm-Balkhed A., Nilsson M., Nilsson M., Dornbusch K., Nilsson L. 2007. multiplex PCR amplification assay for the detection of blaSHV, blaTEM and blaCTX-M genes in Enterobacteriaceae. APMIS 115:1400-1408.

Mazzaroli A., Mammina C., Koncan R., Di Gaetano V., Di Carlo P., Cipolla D., Corsello G., Cornaglia G. 2011. A novel VIM-type metallo-β-lactamase (VIM-14) in a *Pseudomonas aeruginosa* clinical isolate from a neonatal intensive care unit. Clinic. Microbiol. Infec. 17:722-724.

Pitout J., Hossain A., Nancy H. 2004. Phenotypic and molecular detection of CTX-M- β-lactamases produced by *Eschericha coli* and *Klebsiella spp*. J. Clin. Microbiol. 42:5715-5721.

Nordmann P., Poirel L., Carrer A., Toleman A., Walsh T. 2011. How to detect NDM-1 producers. J. Clin. Microbiol. 49:718-721.

Figure S1. Cluster analysis of bacterial communities in the sandy loam (A) and clay (B) soil microcosms. The relationship between the bacterial communities was constructed using an unweighted Unifrac algorithm in QIIME.

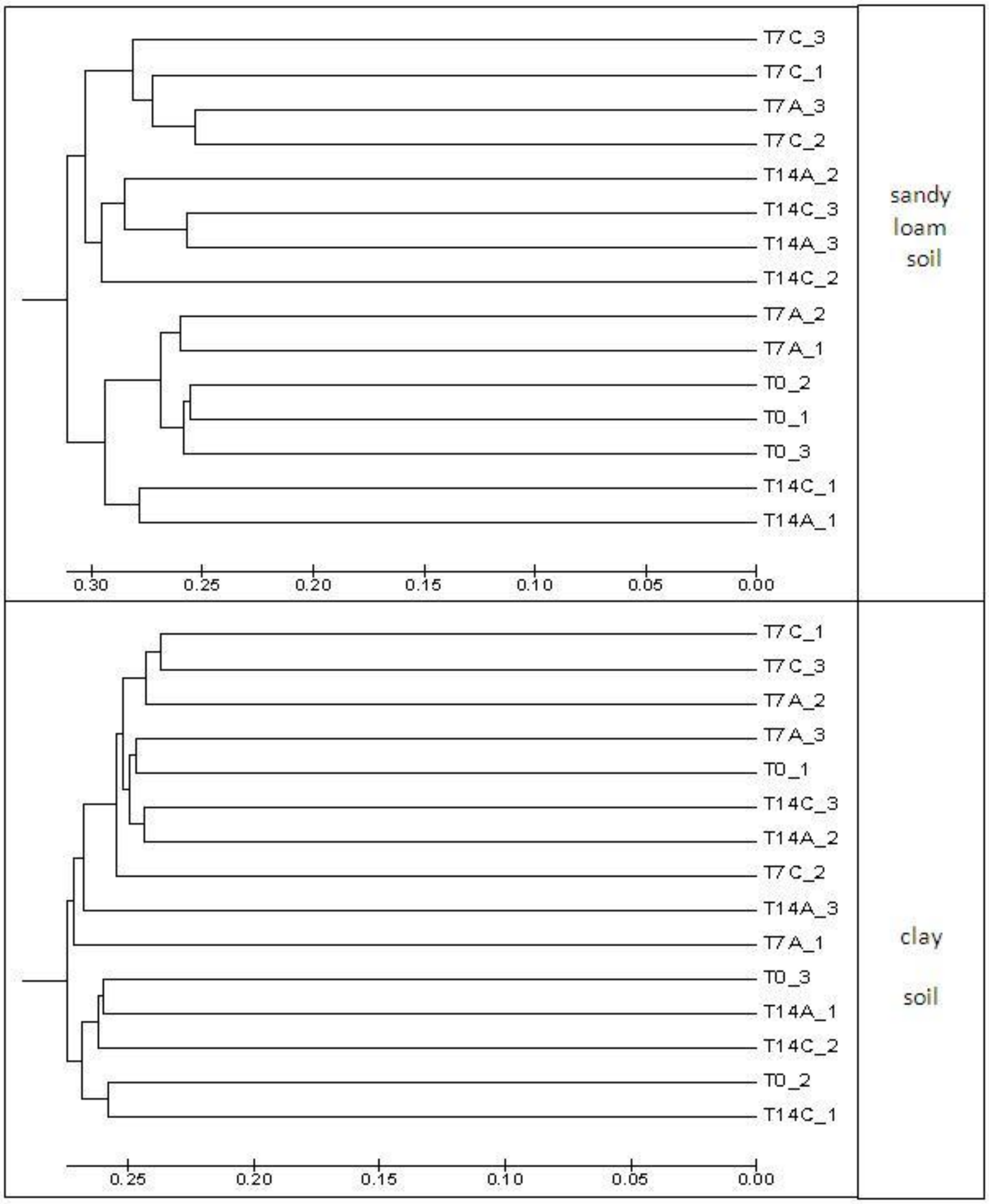

Abbreviations: Non-amended control microcosms (C), antibiotic-amended microcosms (A); experimental initiation (T0), seven days after experimental initiation (T7), 14 days after experimental initiation (T14). Biological repetitions for each treatment are represented by the numbers 1, 2 or 3.

Figure S2. The core microbiome of the sandy loam soil at phylum level.

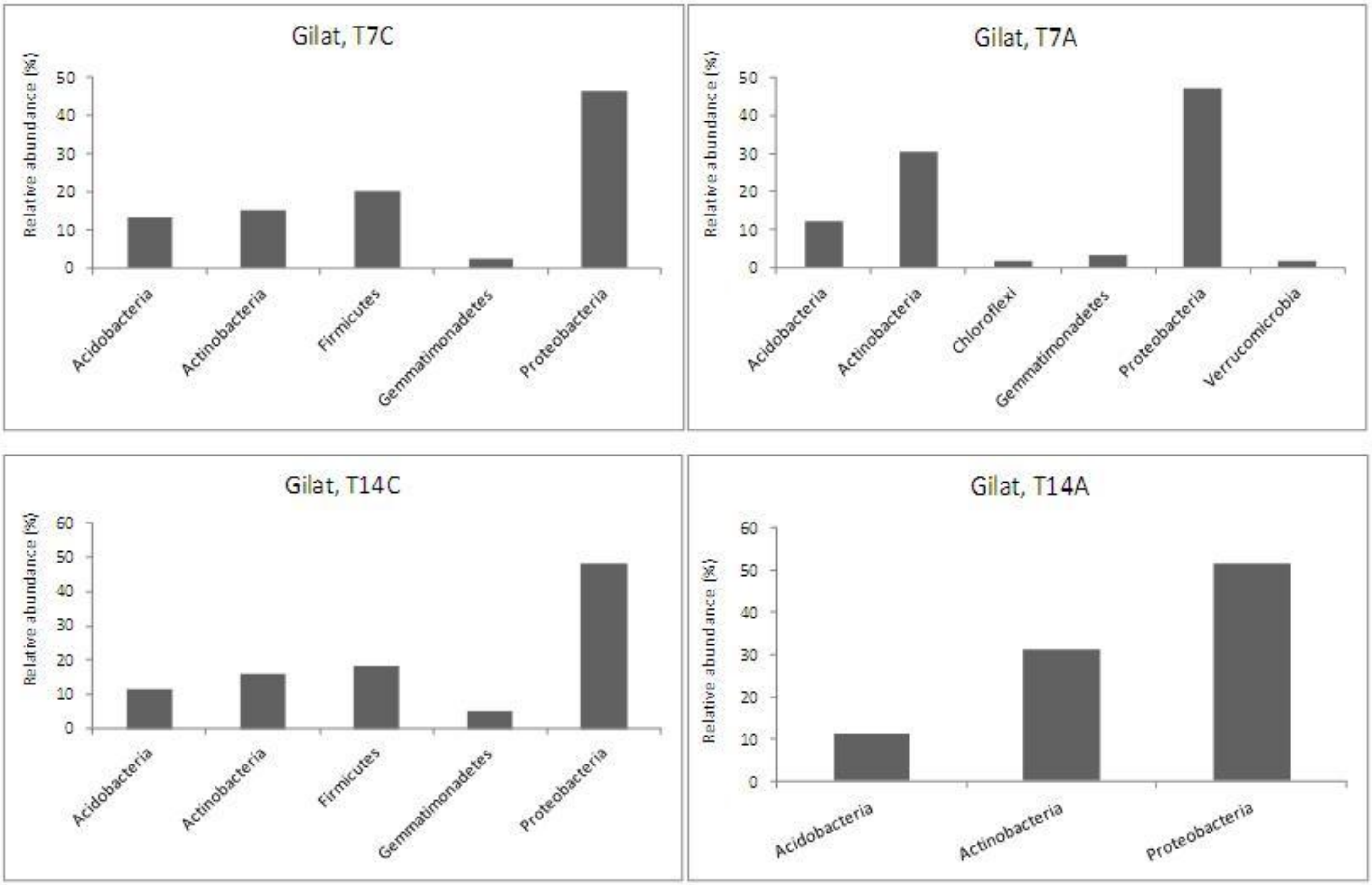

Abbreviations: Non-amended control microcosms (C), antibiotic-amended microcosms (A); seven days after experimental initiation (T7), 14 days after experimental initiation (T14).

Figure S3. The core microbiome of the clay soil at phylum level.

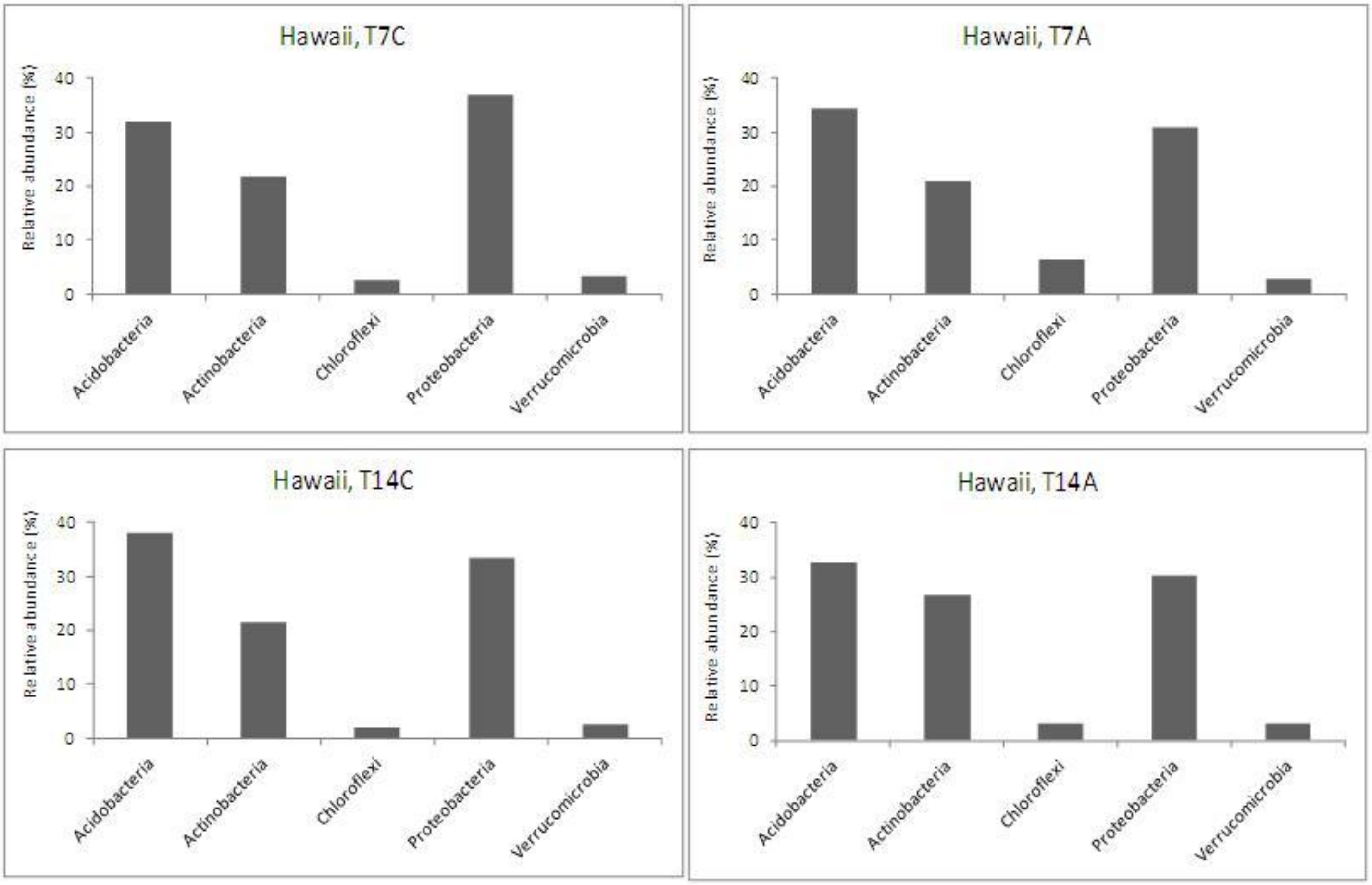

Abbreviations: Non-amended control microcosms (C), antibiotic-amended microcosms (A); seven days after experimental initiation (T7), 14 days after experimental initiation (T14).
